# Refractoriness of Eryptotic Red Blood Cells to *Plasmodium falciparum* Infection: A Putative Host Defense Mechanism Limiting Parasitaemia

**DOI:** 10.1371/journal.pone.0026575

**Published:** 2011-10-21

**Authors:** Paulo Renato Rivas Totino, Cláudio Tadeu Daniel-Ribeiro, Maria de Fátima Ferreira-da-Cruz

**Affiliations:** Laboratory of Malaria Research, Instituto Oswaldo Cruz, Fiocruz, Rio de Janeiro, Brasil; State University of Campinas, Brazil

## Abstract

Recently, we have described that apoptosis-like process of red blood cells (RBC) – eryptosis – in malaria is not restricted to parasitized cells, occurring also in non-parasitized RBC (nRBC). Besides to pathogenic proprieties, apoptosis also participates in the innate defense trough restriction of intracellular pathogens propagation. In the present study, we investigated the capacity of *P. falciparum* parasites to infect eryptotic RBC. Schizont parasitized RBC concentrated by magnetic separation were cultured with eryptotic RBC obtained by ionomycin treatment and, then, parasite growth was evaluated in Giemsa-stained thin blood smears. While parasites infected and developed normally in control non-eryptotic RBC, cultures performed with eryptotic RBC had a marked decrease in parasitaemia. It was noteworthy a great number of free merozoites in eryptotic RBC cultures, indicating that these cells were not susceptible to invasion. We suggest that although eryptosis could be involved in malaria pathogenesis, it could also acting protectively by controlling parasite propagation.

## Introduction

Apoptosis is a physiological process of programmed cell death (PCD) that plays an important role in tissue development and homeostasis as well as in the pathogenesis of different diseases [1]–[3]. Apoptosis has also been implicated in the innate defense against many intracellular pathogens, since infected cells undergo apoptotic process as an altruistic mechanism to prevent infection of adjacent cells and propagation of the pathogen [4]. In spite of this, pathogens have developed diverse strategies to modulate the host cell death pathways assuring, in this way, their intracellular survival and development [5].

In malaria, it has been described that intraerythrocytic development of *Plasmodium* parasites depends on the induction of a suicidal death process in host red blood cell (RBC) similar to apoptosis of nucleated cells, named eryptosis [6]. This process could additionally operate avoiding splenic clearance of parasitized-RBC (pRBC) due to the cytoadherence on microvascular endothelium via phosphatidylserine (PS) [7].

Recently, we have reported that eryptosis in malaria is not restricted to pRBC, as increased levels of non-parasitized RBC (nRBC) eryptosis were observed in *P. yoelii*-infected mice [8]. The importance of this phenomenon to malaria pathogenesis has not yet been assessed, but it is possible that nRBC eryptosis participate in anaemia pathogenesis as well as in complications associated to endothelium cytoadherence as already observed in sickle cell anaemia and sepsis [9], [10]. However, this “pathogenic” effect of eryptosis could also act as a protective mechanism trough restriction of parasite propagation. To address this question, we have investigated the capacity of *P. falciparum* parasites to infect eryptotic RBC.

## Materials and Methods

### Parasite culture

*P. falciparum* parasites (knob^+^ W2 strain) were maintained in continuous *in vitro* culture according to the method described by Trager and Jensen [11]. Parasites were cultured using O^+^ human RBC in RPMI-1640 medium (Sigma) supplemented with 25 mM Hepes (Sigma), 0.2% glucose (Sigma), 23 mM sodium bicarbonate (Sigma), 40 µg/ml gentamycin (Gibco Industries) and 10% heat-inactivated AB^+^ human serum (complete medium). Cultures were maintained at 3% hematocrit at 37°C under an atmosphere of 5% O_2_, 5% CO_2_ and 90% N_2_ (White Martins Praxair Inc).

Synchronization of culture was regularly performed by gelatin flotation [12]. Culture was centrifuged at 300 *g* for 10 min, resuspended in a mixture of 2.4 vol Voluven® (Fresenius) to 1.4 vol of culture medium and, then, incubated at 37°C for 30 min. After incubation, the supernatant containing later stages was collected, centrifuged and cultured with fresh RBC.

### Concentration of parasitized RBC

Concentration of pRBC was performed by magnetic separation, as previously described [13]. Synchronized culture containing 20% of parasitaemia and predominance of schizont was washed by centrifugation at 350 *g* for 10 min and adjusted to 10% hematocrit in complete medium. MACS® columns (25 LD columns, Miltenyi Biotec) were placed in a suitable magnetic support, filled with warmed (37°C) complete medium and, then, 4 mL of culture were applied onto each column. After washing with warmed culture medium, columns were removed from support and, then, schizont pRBC were recovered by adding culture medium and pushing the plunger into the columns. Finally, eluent was centrifuged at 350 *g* for 10 min and schizont pRBC were resuspended in complete medium. The purity of pRBC after magnetic enrichment was around 95%, as observed in Giemsa-stained thin blood smears (data not shown).

### Eryptosis induction

Eryptotic RBC were obtained by treatment with ionomycin. Fresh O^+^ RBC were washed twice in Ringer solution containing (in mM) 125 NaCl, 5 KCl, 1 MgSO_4_, 32 N-2-hydroxyethylpiperazine-N-2-ethanesulfonic acid (HEPES), 5 glucose, and 1 CaCl_2_ (pH 7.4), adjusted to 2% hematocrit in the same solution and, then, incubated for 4 h in presence or absence of 1 µM ionomycin (Sigma). After incubation, RBC were washed thrice and resuspended in complete medium.

Eryptosis induction was evaluated through annexin V staining and cell shrinkage measurement. RBC were resuspended at a density of 1×10^5^ cells/100 µl in annexin-binding buffer (BD Pharmingen), incubated with annexin V-PE (5 µl – BD Pharmingen) for 15 min at room temperature and five times diluted with annexin-binding buffer. RBC were analyzed by flow cytometry (FACScalibur, Becton Dickinson) and forward scatter (FSC), sideward scatter (SSC) and annexin fluorescence (FL-2) were measured.

### Evaluation of parasite growth

Purified schizont pRBC were cultured in fiveplicate in 96-well flat-bottomed plate using ionomycin-treated (eryptotic) or non-treated (non-eryptotic) RBC and complete medium. Cultures were performed at 3% hematocrit and maintained at 37° C under an atmosphere of 5% CO_2_. Parasite invasion and development were estimated by determining percentage of pRBC (parasitaemia) 0, 16, 36 and 60 hours after initiation of culture. Parasitaemia was calculated by light microscopy in Giemsa-stained thin blood smears after counting a minimum of 1000 RBC.

To exclude the possible influence of ionomycin pre-treatment in parasite growth, ionomycin-treated and non-treated RBC were lysed at 3% hematocrit in complete medium by freeze-thawing procedure, centrifuged at 14,000 *g* for 30 min at 4° C and, than, these supernatants instead of standard culture medium were used to perform asynchronous cultures, as described before. Cultures treated or not (control) with 1 µM ionomycin were performed and parasite growth was evaluated after 18 hours of culture.

Images were obtained using a 100x/1.3 oil-immersion objective in an Axioplan 2 microscope equipped with an Axiophot 2 camera (Carl Zeiss).

## Results and Discussion

**Figure 1:**
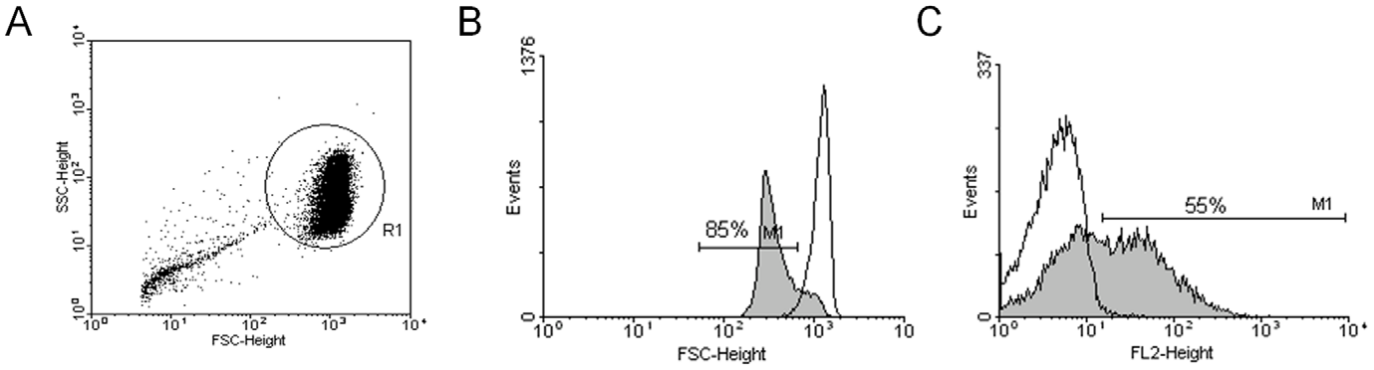
Induction of RBC eryptosis. RBC (A, R1) were incubated at 37°C for 4 h at 2% hematocrit in presence (gray) or absence (black line) of ionomycin (1 µM) and, then, induction of eryptosis was verified by forward scatter measurement (B) and annexin V-PE staining (C).

It is actually known that programmed cell death is not a mechanism exclusive of nucleated cells, occurring also in enucleated cells as RBC. A variety of compounds and pathological conditions inductors of eryptosis – process characterized by Ca^2+^ influx, protease activation, membrane blebbing, cell shrinkage and PS exposure – has been described [14]. In the present study, the capacity of *P. falciparum* parasites to invade and develop into eryptotic RBC was tested using a population of eryptotic RBC obtained through treatment with ionomycin, which was able to induce eryptosis in nearly all treated RBC population (Figure 1).

**Figure 2:**
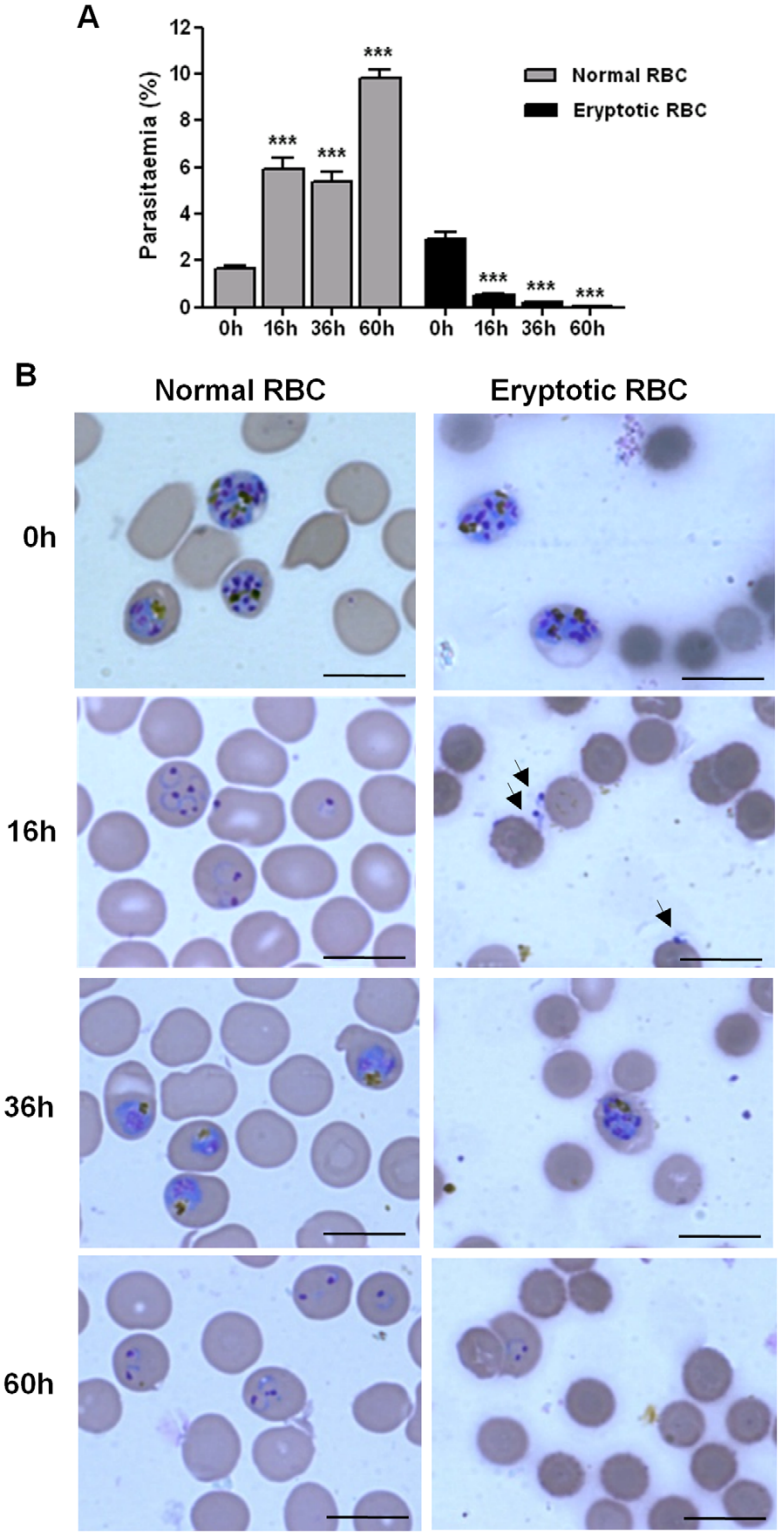
Resistance of eryptotic RBC to *P. falciparum* infection. Schizont pRBC concentrated by magnetic separation were cultured with control non-eryptotic RBC (normal RBC) or eryptotic RBC and, then, parasites invasion (16 and 60 h) and development (36 h) were evaluated in Giemsa-stained thin blood smears. (A) Mean±S.D. of parasitaemia and (B) representative photomicrographs (scale bar  =  20 µm) of the cultures performed in fiveplicate showing: i) eryptotic RBC are refractory to merozoite invasion (16 h); ii) eryptotic RBC are smaller than normal RBC (0–60 h) iii) parasite development in eryptotic RBC culture take place only into normal size RBC (36–60 h) and; iv) eryptotic RBC were still detected after 60 hours of culture. *** indicates significant difference (p<0.001; ANOVA) from initial culture (0 h) in (A) and arrows indicate free merozoites in (B).

**Figure 3:**
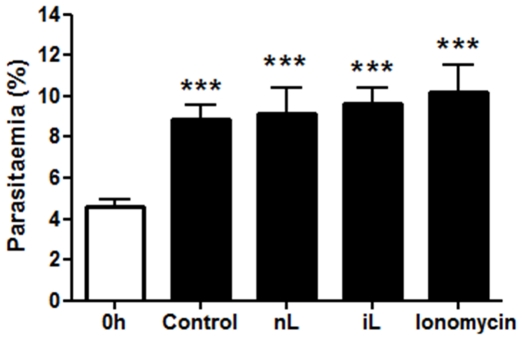
Effect of ionomycin in *P. falciparum* parasite growth. Asynchronous cultures (0 h) were maintained for 18 h in presence of ionomycin or lysates of non-treated (nL) or ionomycin-treated (iL) RBC and, then, parasite growth was evaluated in Giemsa-stained thin blood smears. Non-treated cultures were used as control. Data represent mean±S.D. of parasitaemia of the cultures performed in fiveplicate. *** indicates significant difference (p<0.001; ANOVA) from initial culture (0 h).

As expected, *P. falciparum* parasites infected and developed normally in control non-eryptotic RBC (Figure 2), showing after 16 and 60 h of culture (periods in which parasite invasion was evaluated) a significant increase of 258,5% and 493,6% in parasitaemia, respectively, when compared with initial culture (0 h). Conversely, cultures performed with eryptotic RBC had a marked decrease of 82.3% in parasitaemia after 16 h, reaching 98.9% of reduction at 60 h (Figure 2). It was noteworthy a great number of free merozoites adhered or not to eryptotic RBC after 16 h of culture (Figure 2B), indicating that these cells were not susceptible to invasion. In addition, developing parasites in cultures maintained with eryptotic RBC were only observed in cells showing normal size (Figure 2B – 36 and 60 h), which should correspond to the RBC non-susceptible to ionomycin-induced eryptosis (Figure 1) or those remained after the procedure of pRBC concentration (see material and methods). The presence of developing parasite in ionomycin-treated RBC should indicate that ionomycin pre-treatment did not interfere with parasite viability. Indeed, lysate of ionomycin-treated RBC or, even, ionomycin alone were not able to inhibit parasite growth in culture (Figure 3).

The ability of parasites to infect and develop into apoptotic cells had not been studied so far, possibly, due to the conception that apoptotic cells are rapidly removed by phagocytes through recognition of exposed PS [15]. But, in fact, eryptotic RBC could be target of malaria parasites because these cells have the capacity to adhere on endothelial cells avoiding, therefore, splenic phagocytosis [7], [9]. In the same way, *in vitro* lysis of ionomycin-treated RBC occurs after a period long enough to allow the complete intraerythrocytic development of parasite, as these cells were still detected after 60 hours of culture (Figure 2B). Thus, despite eryptotic RBC could be focus of malaria parasites, in our study, *P. falciparum* parasites were not able to infect eryptotic RBC.

Eryptosis could have opposite key roles in malaria. On one hand eryptosis contributes to complications associated to pRBC sequestration and to anaemia related to splenic phagocytosis of nRBC [6], [8]. On the other hand, this process could reduce parasite levels by splenic phagocytosis of pRBC, controlling parasite growth [16]. In addition, here we disclose another possible protective mechanism of nRBC eryptosis, since these cells are not able to be infected by *P. falciparum* merozoites. This finding could also partially explain why sickle-cell anaemia patients – that usually maintain increased levels of eryptosis [17] – have low levels of parasitaemia [18]. In the same way, the decreased *in vitro* growth of *P. falciparum* in cultures treated with chlorpromazine [19] – a potent eryptosis inductor – could be related to the herein demonstrated inability of parasites to invade eryptotic RBC, since the parasite viability is not influenced by chlorpromazine as well as the protective effect associated to phagocytosis of eryptotic pRBC cannot take place in *in vitro* conditions.

The resistance of eryptotic nRBC to parasite invasion could be attributed to cellular changes occurring during eryptosis. In fact, although the RBC components participating in parasite entry remain poorly defined, it has been shown that two cytoskeletal proteins, i.e. spectrin and actin, which are degraded in apoptosis [20], [21], are involved in host cell infection [22], [23]. Furthermore, a reduction in surface expression of glycophorins – the well-identified receptors to *P. falciparum* in RBC [24] – was also detected in eryptotic RBC from thalassaemia patients [25]. It is possible, therefore, that cell surface and cytoskeleton changes underwent in eryptosis account for the loss of parasite infectiveness observed in our studies, which reinforce the idea that the invasion process of Apicomplexa parasites requires, besides parasite actin-myosin motor, the host cell components [23].

The refractoriness of eryptotic nRBC to *P. falciparum* parasites invasion was firstly herein reported making difficult to deeply discuss the balance between pathogenic and protective effects. In murine models eryptosis of nRBC was reported during anaemia-associated *P. yoelii* 17XL infection [8] or during *P. berghei* ANKA infection when the use of eryptotic inducers was able to enhance eryptosis only in pRBC [26], [27].

In conclusion, we showed that eryptotic nRBC were not target to *P. falciparum* parasites infection and, although eryptosis could be involved in malaria pathogenesis, it could also acting protectively by controlling parasite propagation. Further studies are required to address the magnitude of this phenomenon during malaria infection. In addition, the investigation of eryptosis can provide useful information concerning the host cell components implicated in parasite invasion.
